# Lost in modelling and simulation?

**DOI:** 10.5599/admet.923

**Published:** 2021-03-22

**Authors:** Kiyohiko Sugano

**Affiliations:** Molecular Pharmaceutics Lab., College of Pharmaceutical Sciences, Ritsumeikan University, 1-1-1, Noji-higashi, Kusatsu, Shiga 525-8577, Japan

**Keywords:** physiologically-based pharmacokinetic modelling, scientific literacy, transparency, reproducibility, middle-out approach, structural identifiability

## Abstract

Over the past few decades, physiologically-based pharmacokinetic modelling (PBPK) has been anticipated to be a powerful tool to improve the productivity of drug discovery and development. However, recently, multiple systematic evaluation studies independently suggested that the predictive power of current oral absorption (OA) PBPK models needs significant improvement. There is some disagreement between the industry and regulators about the credibility of OA PBPK modelling. Recently, the editorial board of AMDET&DMPK has announced the policy for the articles related to PBPK modelling (Modelling and simulation ethics). In this feature article, the background of this policy is explained: (1) Requirements for scientific writing of PBPK modelling, (2) Scientific literacy for PBPK modelling, and (3) Middle-out approaches. PBPK models are a useful tool if used correctly. This article will hopefully help advance the science of OA PBPK models.

## Introduction

Over the past few decades, physiologically-based pharmacokinetic modelling (PBPK) has been anticipated to be a powerful tool to improve the productivity of drug discovery and development. Several sophisticated software products have been commercialized since the late 1990s. Plenty of case study reports have been published in peer-reviewed journals, showing nearly perfect prediction, prediction error being much smaller than the variation in the clinical plasma concentration (*C*_p_) - time profile. It seems that we already have achieved a “prediction paradise”[1]. …Really? Recently, multiple systematic evaluation studies independently suggested that the “bottom-up” predictive power of current oral absorption (OA) PBPK models needs significant improvement [2–6]. Almost all case studies had to use parameter optimization on a drug-by-drug basis to fit the simulated plasma concentration (*C*_p_) - time curve to clinical data (Part 3, section 3.4). Expert scientists continue hard experimental works to better understand *in vivo* systems and improve the predictive performance of *in vitro* systems [7–9]. There is some disagreement between the industry and regulators about the credibility of PBPK modelling [10,11]. "Publication bias" (Part 2, section 2.4) and "parameter optimization" (Part 3) have been identified as the main issues of the case studies [11]. Recently, a more realistic view about PBPK modelling has been reported by an industrial consortium [12]. Are we lost in modelling and simulation?

Recently, the editorial board of *AMDET and DMPK* has announced the policy for the articles related to PBPK modelling (Modelling and simulation ethics). This policy is introduced to enhance the science of PBPK modelling. In this article, the background of this policy is explained. This article consists of three parts:

Part 1: Requirements for scientific writing of PBPK modelling,

Part 2: Scientific literacy for PBPK modelling,

Part 3: Middle-out approach (parameter back-calculation from clinical PK data).

The topics discussed in this article have been repeatedly raised before. Transparency and reproducibility (calculation traceability) are critically important for scientific writing (Part 1) [12–14]. From the perspective of evidence-based medicine, case studies are less reliable for assessing the predictive power of a model (Part 2) [15–17]. The issue of parameter identifiability in mathematical modelling has been repeatedly warned in the literature (Part 3) [18–24].

In this article, the above points will be discussed focusing on OA PBPK modelling. But this article would also be beneficial to the other PBPK models. *PBPK models will be a useful tool if used correctly*. *This article will hopefully enhance the science of OA PBPK modelling in the future.*

## Part 1: Requirements for scientific writing of PBPK modelling

The policy on the scientific writing of physiologically-based pharmacokinetic modelling (PBPK) articles complements the current journal's author guidelines that cover *in vivo* and *in vitro* methods based on scientific literacy. This guideline is also in line with regulatory guidance for industry regarding PBPK modelling [12–14]. In this article, past articles that do not comply with this policy are not quoted, because it would be disadvantageous for the authors. However, readers will see that the majority of the past papers have some issues raised in this article.

Transparency and reproducibility are mandatory to ensure the credibility of PBPK modelling. As sciences and technology progress, model equations and physiological data can be updated in the future (section 2.1). Therefore, the use of the current best estimate parameter in PBPK modelling is appropriate. However, if the physiological data or model equation changes in the future, past articles need to be re-evaluated. For example, a wide variety of small intestinal fluid volume (*V*_si_) has been used in oral absorption (OA) PBPK modelling, ranging from less than 100 mL to over 1500 mL (the former is based on the recent MRI measurements) [25–29]. Despite more than a 15-fold difference, they all claimed good predictability (section 3.3). This cannot be true. If all details had been reported, we can trace the calculation and retrospectively inspect the reason for this contradiction. But if there was an undisclosed part, it is not possible to judge whether the past good prediction is just a lucky coincidence or due to other reasons. When such an inconsistency arises in regulatory submissions, it causes a more troubling situation. To ensure the credibility of PBPK modelling, authors must write the manuscript as transparent as possible to enable inspection by peer-reviewers and ensure reproducibility by independent third parties.

### 1.1. Introduction section

The purpose of PBPK modelling should be explained in the introduction section. The question of interest and the context of use (COU) for PBPK modelling should be described [10]. There is no one-size-fits-all model (section 2.8). A mathematical model that suits the purpose should be selected. The reason for selecting a PBPK model should be explained.

### 1.2. Method section

Based on the scientific ethics of transparency and reproducibility, authors are requested to disclose the model equations, physiological parameters, and drug parameters (section 2.1) that are sufficient for peer-review (or appropriate references to them). Undisclosed parts cannot be peer-reviewed in the first place. In addition, according the rule of science, an experimental section should be written as detailed as possible so that to “enable” reproducing the same result. This point is critical for ensuring the credibility of science. If the word "reproducibility" is interpreted literally, even if the model equations and parameters are not disclosed (as in a black box model), the results can be reproduced by using the same input data and the same software (same version). In the context of scientific credibility, for the articles of mechanistic models (including PBPK models (section 2.1)), it should be taken as calculation traceability. A method section should be detailed enough to enable someone who wants to trace the calculation process, at least in the essential parts for COU. If a part of the model is not disclosed, that part becomes a "black box”. Because a black box can mask errors in any part of the model, the credibility of the entire research is damaged by the existence of only one undisclosed part.

#### The scientific validity of a research article is the author’s responsibility, even when using commercial software.

In papers using commercial software, the model equations and physiological parameters are often described as "default”. The default information may have been disclosed in a user’s manual. However, it is not available for peer-reviewers and readers. The default model equations and physiological parameters must also be publicly disclosed.

In nearly all case study reports of OA PBPK modelling, case-by-case parameter optimization (back-calculation) has been committed to fit the simulated plasma concentration (*C*_p_) - time curve to the clinical observation on a drug-by-drug basis (called “local middle-out approach”, see Part 3). Any parameter back-calculation from clinical PK data must be explicitly stated in the method section. Case-by-case parameter back-calculation could have been unconsciously committed. For example, *ad hoc* selection of estimated permeability values from *in silico*, *in vitro*, or *in situ* data is a kind of parameter back-calculation. Various terms implying a subtle adjustment such as “optimize”, “fit”, “adjust”, “recover”, “refine”, and “software estimated”, have been used to refer to parameter back-calculation. However, the difference from the initial value often exceeds two-fold due to the large errors in *in vitro* – *in vivo* extrapolation (IVIVE) and/or *in silico* prediction [30,31] (section 3.10).

When a parameter is calculated from a chemical structure by an *in silico* model, the prediction accuracy of the in silico model should be shown (or referenced) (section 3.8 (iv)).

### 1.3. Results section

**Figure 1.**
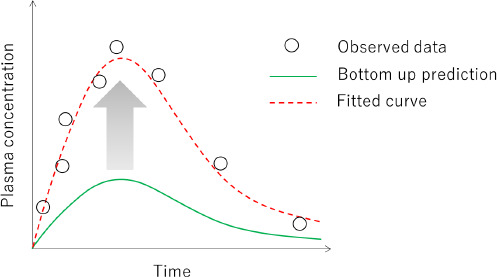
Schematic illustration of simulated and observed *C*_p_ – time profiles

When case-by-case parameter back-calculation was committed, the initial input parameters and the simulation results *before parameter back-calculation* must be reported in the result section (Figure 1). Unfortunately, the failed prediction is often undisclosed in case study reports. However, this information is important for evaluating the creditability of a back-calculated parameter. For the advancement of science, failed predictions are just as (or even more) important than successful cases (section 3.4).

After case-by-case parameter back-calculation, the fitted curve must be labeled as “fitted” (NOT “predicted”) for the same clinical PK data used for parameter fitting. The fitted *C*_p_ - time curve is not a predicted curve, because the observed *C*_p_ - time profile has been used to back-calculate the parameter (self-referencing) (section 3.11).

In oral absorption (OA) PBPK modelling, the fraction of a dose absorbed (*F*_a_) is one of the most important outputs for understanding the oral absorption of a drug. Although clinical *F*_a_ data (or its surrogates (section 2.6)) is not always available for model validation, simulated *F*_a_ data is important for interpreting the simulation results regarding the oral absorption of a drug, at least for biopharmaceutics and formulation scientists (section 2.2). Therefore, a simulated *F*_a_ value should be reported. The simulated *F*_a_ value (or the *F*_a_ – time profile) is available as an output in all commercial OA PBPK software products. However, unfortunately, this value has not been reported in many reports.

### 1.4. Discussion section

When parameter back-calculation from clinical PK data is committed, the following points should be discussed (See Part 3 for details) [18–24,32–34]: (i) the reason for the mismatch of the initial “bottom-up” prediction and clinical PK data, (ii) the reason for selecting a parameter as the subject of back-calculation (sections 3.5 to 3.7), (iii) parameter identifiability (section 3.2), (iv) the accuracy of the other parameters than the subject of parameter back-calculation (section 3.8), (v) the plausibility of back-calculated parameter considering physicochemical properties, *in vitro* data, and *in vivo* physiology (section 3.10), (vi) the constancy of the back-calculated parameter in the clinical and/or population conditions (COU) to be predicted (sections 3.3 and 3.13), (vii) the validity of the optimized model (section 3.11).

### 1.5. References

The above policy applies to the references. The authors should cite reliable articles as references.

## Part 2: Scientific literacy for physiologically-based pharmacokinetic modelling

In Part 1, we discussed how to write a scientific article for PBPK modeling. In this part, we will discuss how to interpret PBPK papers and how to evaluate PBPK models. Physiologically-based pharmacokinetic (PBPK) models are not as easy to use as a smartphone app. As mentioned in the introduction, commercial PBPK software may not be so perfect as a user might believe [2–6,11,35,36]. Before using a PBPK model, we must understand *the scientific literacy for mathematical modelling*. In this part, the scientific literacy required for PBPK modelling is discussed. We are fully aware that there are various opinions among the modelers on this topic. The purpose of this part is to suggest several viewpoints when using a PBPK model.

### 2.1. The basic concept of physiologically-based pharmacokinetic models

**Figure 2.**
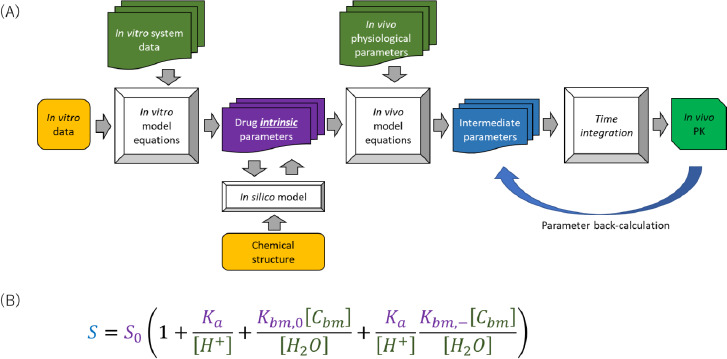
Overall prediction scheme of PBPK modelling (A) and the solubility model in biorelevant media for an acidic drug (B). (A) *In vitro* data is first reduced to drug intrinsic parameters using the mathematical model of an *in vitro* system [38]. The drug intrinsic parameters are then converted to *in vivo* PK profiles via intermediate parameters (e.g., the permeation rate constant (*k*_perm_)). The drug intrinsic parameters are directly related to a chemical structure so that suitable for *in silico* prediction and drug design. (B) Drug intrinsic parameters: purple, physiological parameters: green, and intermediate parameter: blue [39,40]. *K*_a_ is the dissociation constant, *S*_0_ is the intrinsic solubility of a drug, and *K*_bm,0_ and *K*_bm*,-*_ are the bile micelle partition coefficients for unionized and anionic drug molecular species, respectively. [*C*_bm_] is the bile micelle concentration, [H^+^] is the proton concentration (= 10^-pH^), and [H_2_O] is the concentration of water.

Basically, a PBPK model consists of independent *a priori* information of drug and formulation parameters, physiological and biological parameters at the organ level, and model equations [37]. All model equations and parameters have a physical and physiological basis (mechanistic basis) (Figure 2). PBPK models provide a mechanistic representation of pharmacokinetics and allow *a priori* “bottom-up” prediction of *in vivo* PK profiles from *in vitro* data for various clinical situations. To simulate the effect of a physiological factor, the model equation must include the factor as a system parameter. As drug parameters, the parameters that are intrinsic to a drug should be used. By combining drug-intrinsic parameters with physiological factors in model equations, the effects of physiological conditions can be simulated. This model structure allows the PBPK model to handle population variability and physiological covariates. Furthermore, the oral absorption (OA) PBPK model can account for the bioequivalence of various formulations considering confidence intervals. This point is one of the specific features that can be handled by PBPK modelling.

An OA PBPK model consists of the model equations of solubility, dissolution rate, permeability, etc. Therefore, it is important to understand these equations before using PBPK modelling (section 1.2, 2.3, and 2.7). For example, in a physiologically-based solubility model (Figure 2B), the pH and the bile micelle concentration (*C*_bm_) are used as physiological parameters, and the intrinsic solubility (*S*_0_), p*K*_a_, and bile micelle partition coefficients (*K*_bm_) (for each unionized and ionized drug molecules) are used as drug parameters [35,39,40]. Physiologically-based dissolution [41–48] and permeation [49–55] models have already been reported in the literature and implemented in some OA PBPK models. The followings are examples of physiologically-based dissolution and permeation models in the simplest form.

#### Dissolution model (for mono-dispersed spherical small particles) [41–48,56]



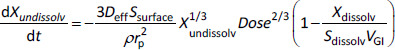



where *X*_undissolv_ and *X*_dissolv_ are the amounts of a drug undissolved and dissolved in the gastrointestinal fluid, respectively. *D*_eff_ is the effective diffusion coefficient, *Dose* is the dose strength, *ρ* is the true density of the drug substance, and *r*_p_ is the particle radius (*r*_p_ < 30 μm). *S*_surface_ and *S*_dissolv_ are the solubilities of a drug at the particle surface and in the bulk fluid, respectively. *V*_GI_ is the gastrointestinal fluid volume. The dissolution rate constant (*k*_diss_) is *k*_diss_ = 3 *D*_eff_ *S*_surface_*/(ρr*_p_*^2^). S*_surface_, *S*_dissolv_, and *D*_eff_ are affected by physiological factors such as pH, buffer capacity, bile micelle concentration (Figure 1(b)).

#### Permeation model (for passive diffusion) [49–55]



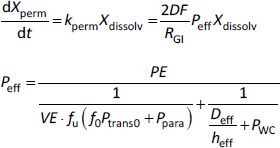



where *X*_perm_ is the amounts of a drug permeated the intestinal wall, *k*_perm_ is the permeation rate constant, *DF* is the degree of flatness of the small intestinal tube, *R*_GI_ is the radius, *P*_eff_ is the effective intestinal membrane permeability, *PE* is the plica expansion factor, *P*_UWL_ is the UWL permeability, *VE* is the villi expansion factor, *f*_u_ is the fraction of unbound drug molecule species (free fraction), *f*_0_ is the fraction of undissociated drug molecule species (calculated from p*K*_a_ and pH), *P*_trans0_ is the intrinsic passive transcellular permeability, *P*_para_ is the paracellular permeability, *h*_eff_ is the effective thickness of the UWL, and *P*_WC_ is the water conveyance.

Instead of using a physiologically-based mechanistic dissolution model, an experimental *in vitro* dissolution profile can be plugged into an OA PBPK model as a kind of intermediate parameter (Figure 2A)[57]. This strategy is often used when the dissolution process is the key determinant of oral drug absorption. In this case, the dissolution profile is pre-fixed so that it is not altered by the physiological factors in the computer simulation. For example, when a dissolution profile measured at pH 1.2 is plugged into an OA PBPK model, it is not changed even if the gastric pH value is changed from pH 1.2 to pH 5.0 in the OA PBPK model. Consequently, it cannot account for the effect of inter- or intra- subject variability of relevant physiological parameters on drug dissolution processes. Such a simulation strategy may be understood as a variation of the convolution method, rather than “physiologically-based” computational modelling (in the case of using *in vivo* predictive dissolution testing [58–61], the *in vitro* dissolution profile itself is physiologically-based).

### 2.2. To Explain or to predict? Which is the position of a PBPK model?

The purpose of mathematical modeling is not only to calculate the predicted values, but also to explain the observed and predicted data (understanding, interpretation) [62]. The interpretability of a model is especially important for medical applications. For an explanation-oriented model, ideally, all system parameters should be set based on independent *a priori* information. From the viewpoint of mathematical modelling, PBPK models are more of an explanation-oriented model when compared to more complex statistic models like deep learning [63]. In general, the interpretability of a model decreases as the model becomes more complex. Simple models have a great value of “interpretability” by themselves [64]. On the other hand, complex models may (or may not) show better predictive power (prediction performance, generalization performance) (section 2.8).

**Figure 3.**
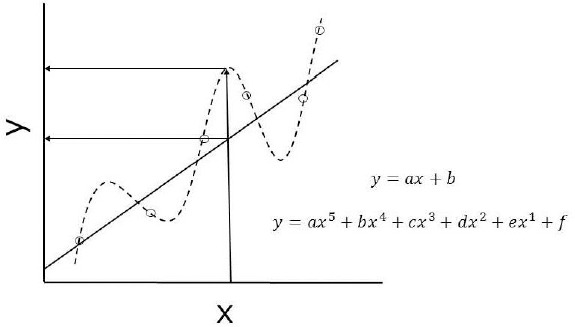
Overfitting.

The predictive power and description capability of a model are different. The description capability of a model is to describe existing data, whereas the predictive power is to predict unknown data. As a model becomes more complex and flexible, description capability always increases. However, predictive power does not always increase, because assumptions and errors can accumulate with the increasing complexity of a model (sections 2.8 and 3.10). When parameterization is used, a complex model is often prone to overfitting (Figure 3).

In mathematical modelling, generally speaking, to compensate for its lesser interpretability and its higher risk of overfitting, *a complex model should show a significant advantage over a simple model* *with respect to predictive power for the context of use* [20,64–67]. If simple and complex models show comparable predictive power with regard to the context of use, the simple model should be selected (cf. Occam’s razor, the principle of parsimony) [68]. At the same time, when a complex model shows better predictive power, the complex model should be selected (section 2.8).

In Figure 3, the quintic equation perfectly describes (fits) the data. If the experimental data is error-free, it is suitable for predicting unknown Y from new X data. In practice, especially for biological data, there is always error in experimental data. For objective model selection, statistical indices such as the Akaike Information Criterion (AIC) can be used [69].

### 2.3. How to interpret a model

There are several ways to interpret a mathematical model to understand the behavior of the system of interest.

#### Parameters

The first step to understanding a PBPK model is to know the parameters that affect the physical and physiological processes. Parameters that are not included in the model equation cannot be captured by the model. Before using a PBPK model, we must understand the key factors that affect the pharmacokinetic processes of a drug (sections 2.1 and 2.7). Intermediate parameters can be used to understand the contribution of each process.

#### Mathematical structure

A mathematical structure represents the relationship between the system parameters, the dependent variables, and the independent variables, for example, being additive, synergistic, proportional, inversely proportional, exponential, etc. This point is important for parameter identifiability (see Part 3).

#### Rate-limiting step

In any kinetic model, it is essential to understand the rate-limiting step (or rate-limiting parameter) [70,71]. For example, the rate-limiting step that determines *F*_a_ (FaRLS) can be diagnosed from the solubility, dissolution, and permeation numbers [53,72]. From FaRLS, the sensitive parameters for *F*_a_ of a drug can be easily identified. The rate-limiting step of intestinal membrane permeation can be diagnosed by using a physiologically-based permeability model [73,74]. The rate limiting step could differ depending on the physiology of each subject.

#### Parameter sensitivity analysis

Parameter sensitivity analysis (PSA) has been utilized to interpret black-box models, such as deep learning. PSA could also be useful in PBPK modelling. In PBPK modelling, PSA has been generally used to help deciding which parameters require further consideration either for additional *in vitro* measurement or parameter back-calculation (section 3.5). A PBPK model includes all parameters and models, however some of these may have little or no influence on the outcome.

### 2.4. Evidence level regarding the predictive power of PBPK models

The predictive power of a mathematical model can be evaluated based on the concept of evidence-based medicine (EBM) [15–17]. According to EBM, the evidence level of a case study is low. *Case studies are prone to publication bias.* Successful cases tended to be published whereas failed cases are usually not published. Historically, publication bias has caused the illusion of knowledge, especially in the medical area.

A systematic study is essential to evaluate the predictive power of PBPK models. For the bottom-up prediction by OA PBPK models, several systematic evaluation studies have already been published [2–5]. However, middle-out approaches (see Part 3) have been evaluated mostly by case studies (or the collection of case studies). Systematic evaluation is required for middle-out approaches *using a standardized procedure* (more precisely, for local middle-out dynamic PBPK models) [10,75,76].

Although the evidence level of a case study is limited, it is still beneficial as far as appropriately conducted and reported. However, the creditability of case studies has often been compromised by the existence of a black box in a model (see Part 1) and inappropriate parameter optimization (see Part 3) [11]. The middle-out approach is discussed in detail in Part 3.

### 2.5. Points to consider for systematic evaluation of predictive power

*Dataset:* In OA PBPK modelling, the biopharmaceutical classification system (BCS) can be a good starting point [6,77,78]. A test set biased to BCS class I drug (high solubility/high permeability) should be avoided, because complete absorption is easily expected for BCS class I drug. Similarly, no food effect and no gastric pH effect on *F*_a_ are expected for BCS class I drugs (but they could affect *C*_max_ and *F*). In the case of BCS class I drugs, complex OA PBPK models may add little advantage over simple models for *F*_a_ prediction (depending on the purpose of the model) (section 2.8).

**Figure 4.**
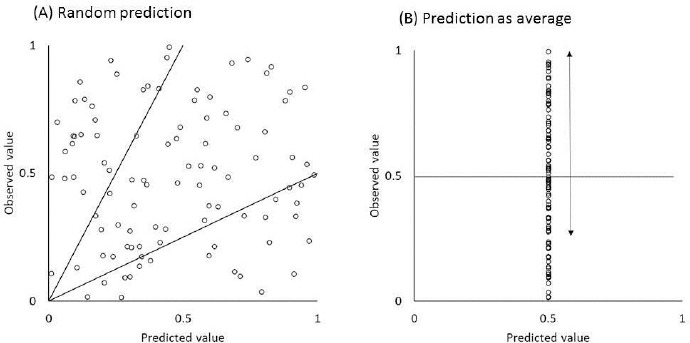
Naïve prediction. (A) Random prediction, (B) prediction by average. The percentage within a two-fold error is 50 % for (A) and 75 % for (B).

*Control:* A simple model or a naïve prediction (Figure 4) should be used as a control to evaluate the predictive power of complex models [5,50,51,79].

*Outcome:* The absolute bioavailability (*F*) or *F*_a_ has been used to evaluate OA PBPK models [2]. *F* is calculated by subtracting the effects of *Dose* and systemic clearance (*CL*) from oral AUC data using i.v. data (*F* = (AUC_p.o*.*_/*Dose*_p.o*.*_)/(AUC_i.v*.*_/*Dose*_i.v*.*_)). *F*_a_*F*_g_ can then be calculated from the hepatic clearance (*CL*_h_) as *F*_a_*F*_g_ = *F/F*_h_, *F*_h_ = 1 – *CL*_h_ /hepatic flow [80] (*F*_g_: the fraction escaping the intestinal wall metabolism, *F*_h_: the fraction escaping the hepatic first-pass metabolism). In addition, there are several methods to estimate *F*_a_ from clinical PK data (section 2.6) [5,81]. AUC is less suitable for evaluating the predictive power of OA PBPK models because the main determinant of AUC is *Dose* and *CL* in many cases.

*Statistics*: Statistics must be carefully interpreted. The percentage within a two-fold error is often used to evaluate the predictability of PBPK models. However, even when the predicted and observed *F* values are randomly distributed from 0 to 1, the percentage within a two-fold error becomes 50 % (Figure 4A) (see also Figures 3 and 4 in [6]). When predicted as average, 75 % is within a 2-fold error (Figure 4B). This kind of prediction is called “naïve prediction”. This percentage increases when the data set is biased towards BCS class I.

### 2.6. Surrogates of F_a_ data

In OA PBPK modelling, the fraction of a dose absorbed (*F*_a_) is an important simulation output (section 1.3). However, there is no exact method to measure *in vivo* *F*_a_. Therefore, one or a few approximations have been used to estimate *F*_a_ from *in vivo* PK data [50,70,79,82–84].

- Mass-balance data using a radio-labeled drug for i.v. and p.o.
- From absolute bioavailability (*F*), hepatic clearance (*CL*_h_), and the hypothesis of *F*_g_ = 1 (section 3.8 (v)).
- Relative bioavailability of solution vs. solid formulation.
- Relative bioavailability in the fasted state vs. the fed state (when *Do* < 1 in the fed state) (*Do*: the dose number (*Dose*/ (*S* ×*V*_si_)), *S*: solubility, *V*_si_: small intestinal fluid volume).
- Relative bioavailability between the dose strengths where *Do* < 1 and *Do* > 1 (AUC can be corrected by elimination *T*_1/2_ for nonlinear clearance drugs).

The mass-balance data would be the most reliable data to estimate *F*_a_. However, these data are rarely available. When i.v. data is available, we can estimate *F*_a_*F*_g_ from *F* and *CL*_h_. When i.v. data are not available, for high permeability drugs, the relative bioavailability to an oral formulation that eliminates the effect of solubility and dissolution ((C)-(E)) can be used as a surrogate of *F*_a_ (cf. in this case, the effects of *F*_g_ and *F*_h_ are canceled out). The permeability category (low/high) can be reliably diagnosed by *in vitro* permeability assays [85]. In the case of high *P*_app_ drugs (> metoprolol *P*_app_ at pH 6.5, metoprolol log *D*_pH6.5_ = -1.1)[86], *F*_a_ will be greater than 0.8 with a very high probability when there is no solubility and dissolution limitation. Based on this high reliability, Caco-2 *P*_app_ data has been used for regulatory biowaiver submission (note: for highly lipophilic drugs (log *D*_pH6.5_ > 1.5), the Caco-2 assay may underestimate the permeability of the drug due to experimental artifacts such as membrane binding [87,88], leading to a misassignment of a drug as low permeability (this can be identified by a mass balance study in Caco-2). In this lipophilicity range, the diffusion through the unstirred water layer becomes the rate-limiting step of *in vivo* membrane permeation. Therefore, a rough estimation of permeability from experimental log *D*_pH6.5_ will be sufficient and could be more reliable [55,86]).

The above methods showed similar *F*_a_ values when applied to the same drug in most cases [50,79,82,83], confirming the validity of these methods. More than 600 clinical *F*_a_ data has been compiled from the literature and has been used to evaluate OA PBPK models [5,50,73,79,83,86,89,90]. For low *F*_a_ drugs the inter-subject variability is usually very high, and a clinical study may be an unrepresentative sample of the population.

### 2.7. Often forgotten physicochemical mechanisms in OA PBPK modelling

A good understanding of physicochemical processes in oral drug absorption is specifically important for OA PBPK modelling (see conclusion part). Below are some examples of physicochemical mechanisms that are often forgotten in OA PBPK modelling.

#### Example 1: *P*_eff_ estimation from *in vitro* data

It is well known that the effective intestinal membrane permeability (*P*_eff_) is markedly affected by bile micelle binding [51,91–94], the unstirred water layer (UWL) [74,95–98], and the anatomical features (villi and fold structures) [99] (section 2.1) (cf. the *P*_eff_ value includes the free fraction effects by definition in most cases of commercial software). However, these factors cannot be captured by an empirical equation of *P*_eff_ = *aP*_app_^*b*^ (log *P*_eff_ = *A’* + *B’*log *P*_app_) (*P*_app_: *in vitro* apparent permeability). This empirical equation cannot correctly explain and predict the food effect (especially the negative food effect) [51,91,100], the formulation effect (solubility-permeability trade-off)[101,102], and species differences [52,103]. The empirical coefficients, *a* and *b* (or *A’* and *B’*), are usually determined by the regression analysis between *P*_eff_ and *P*_app_ using hydrophilic model drugs (log *D* < 1)[31]. Therefore, lipophilic drugs are beyond the applicable range of the standard curve. In addition, *P*_app_ is usually measured in the absence of bile micelles on the apical side and albumin on the basolateral side. The thick UWL in the *in vitro* systems mask the membrane permeability when not rigorously agitated [98,104]. Finally, and most importantly, this equation is an empirical correlation so that it should not be referred to as “physiologically-based”*.* A mechanistic physiologically-based permeation model that considers these factors is available in the literature (section 2.1) [49–55] and in some commercial software products.

#### Example 2: Dissolution in the stomach

The gastric fluid is acidified by hydrochloric acid (HCl). Because HCl is not an efficient buffer, the pH value of the gastric fluid increases when a free weak base drug is dissolved [105]. In addition, the solid surface pH is markedly increased by the dissolving free base molecules [42,43,48,61,106,107]. Therefore, the solid surface solubility (*S*_surface_) and the bulk phase solubility (*S*_dissolv_) must be differentiated in the Nernst-Noyes-Whitney equation (section 2.1) [35]*.* These two factors are important for predicting the effect of gastric pH on drug absorption. A mechanistic dissolution model that considers these factors is also available in the literature (section 2.1) [42,43,48,61,79,106,107] and in some commercial software products.

#### Example 3: Salt dissolution

The dissolution modelling of a salt form drug is not that easy as one might imagine. The solid surface solubility of a salt is significantly higher than that of the free form (> 100-fold in most cases). However, the equilibrium solubility in the pH-controlled region becomes the same regardless of the starting material is a free form or a salt form (unless the residual solid (equilibrium maker) show different solid forms) (cf. a salt form coverts to a free form in the pH-controlled region). In drug discovery and development, the solubility of a drug substance is usually measured in well-buffered media after a sufficient incubation time to achieve equilibrium [108–111]. The small intestinal pH (about pH 6.5) is in the pH-controlled region in most cases. In addition, a salt form may or may not show faster dissolution and more importantly supersaturation after dissolution, because the solid surface precipitation of a less soluble free form can inhibit the dissolution of its salt [112,113]. The mechanism of supersaturation and precipitation is not well understood at this moment. However, at least, it does not simply follow the first-order kinetics [114–119].

In addition, *in silico* models for the physicochemical properties of a drug are not so accurate as to be used for PBPK modelling [120]. Solubility measurements are not as easy as a modeler might imagine [109,111,121]. The p*K*_a_ values change between 25 °C and 37 °C [122,123]. Enabling formulations such as amorphous solid dispersion [124], co-crystal [125,126], nanoparticles [127–129], and self-emulsifying drug delivery system [130,131] also requires an in-depth understanding of physical chemistry for OA PBPK modelling. Further investigations on these points are required in the future (see conclusion part).

### 2.8. A simple PBPK model or a complex PBPK model, which one to use?

***“Everything should be made as simple as possible, but not simpler.”***

Albert Einstein

As mentioned above, we are fully aware that there are various opinions among the modelers on the selection of mathematical models. A PBPK model should be selected considering the purpose of modelling and available data at each drug discovery and development stage. In the early drug discovery stages, a simple PBPK model may be sufficient. In the late drug development stages and after launch, a complex PBPK model may be required to investigate more complex clinical situations.

For the use in the early drug discovery stages, simple models can show sufficient prediction performance [20,64,82,132,133]. A simple OA PBPK model has shown good prediction performance for the fraction of a dose absorbed (*F*_a_) [5,50,79,83,134] and plasma concentration (*C*_p_) - time profiles [135–143]. For relative bioavailability (*F*_rel_) prediction, a simple OA-PBPK model is also available for the food effect (via bile micelles) and gastric acid effect predictions [51,79,100]. By multiplying AUC in the fasted state (or low gastric pH) with *F*_rel_ (i.e., AUC ratio), AUC in the fed state (or high gastric pH) can be estimated. Minimal PBPK models have also been proposed to reduce the complexity of a model (see section 3.13 for metabolic DDI prediction) [21,144].

To explain and predict complex pharmacokinetics and population variation in late drug development and product life-cycle management, a PBPK model should have sufficient components. Commercial software products that implements a complex dynamic OA PBPK model would be especially useful for these purposes.

## Part 3: Middle-out approach

Part 2 discussed the interpretation and evaluation of the PBPK model. One of the most difficult parts of evaluating a PBPK model is the validity of the middle-out approach. Therefore, middle-out approaches are discussed in this part. Middle-out approaches have been widely utilized in real drug discovery and development [21]. They would be effective to improve various processes in drug development. At the same time, if they are inappropriately used, they could cause trouble. The purpose of this section is to discuss how to properly use the middle-out approach in OA PBPK modelling.

### 3.1. The concept of the middle-out approach

In a middle-out approach, some parameters of a PBPK model are derived from *in vitro* data (bottom-up), while others are derived from clinical PK data (top-down) *on a drug-by-drug basis* (see also section 3.14). In the following sections, this prediction scheme is referred to as a “local (drug-by-drug)” middle-out approach to differentiate it from a “global” middle-out approach (section 3.15) [145]. For drug-drug interaction (DDI) prediction, the local middle-out approach *using a CYP specific inhibitor* *or substrate* has been successfully used [21] (section 3.12). However, it has been pointed out that the inappropriate use of a local middle-out approach reduces the creditability of OA PBPK modelling [17,20].

**Figure 5.**
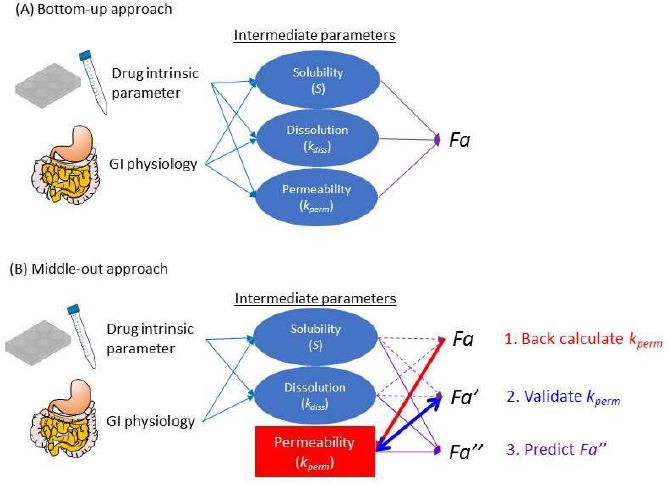
Bottom-up and middle-out approaches for OA PBPK modelling. A middle-out approach brings an empirical model into a PBPK model (the red line and square). For parameter back calculation and validation, separate clinical data (*F*_a_, *F*_a_’) must be used.

A middle-out approach brings an empirical model into PBPK modelling (Figure 5, the red line and square). Therefore, we should follow the good practice of empirical modelling: (i) Before back-calculation, *parameter identifiability must be carefully examined* (sections 1.4 and 3.2) [18–23,32,33]. (ii) The degree of freedom must be enough to avoid overfitting (section 2.2, Figure 3). (iii) The predictive power must be validated using clinical data that is not used to back-calculate the parameter (called “cross-validation”) (section 3.11). (iv) The optimized empirical model should be used within the parameter space limited by the data used for back-calculation (so that as interpolation) (This is a general recommendation for empirical models. For the hybrid of empirical and mechanistic models, this point (iv) needs further in-depth discussion.).

### 3.2. Parameter identifiability: a simple explanation

The issue of parameter identifiability in biological mathematical modelling (including PBPK modelling) has been repeatedly warned in the literature [18–24]. However, this issue seems to have been overlooked in many case studies using a local middle-out approach. In this section, parameter identifiability is plainly explained focusing on OA PBPK modelling.

In a middle-out approach, the identifiability of a parameter(s) must be assessed before back calculating the parameter, whether the parameter(s) can be uniquely and reliably identifiable from the input-output data [18–24]. There are two types of parameter identifiability: statistical and structural. Statistical identifiability is related to the experimental error of the observed data. However, even with error-free data, a model parameter could be structurally non-identifiable.

To illustrate the concept of structural identifiability, let’s consider an equation of *Y* = *abX*, where *X* is an explanatory variable, *Y* is an object variable, and *a* and *b* are system parameters [23]. The lump quantity *a*·*b* is uniquely identifiable from *X* and *Y*, while the individual parameters of *a* and *b* are non-identifiable (it is mathematically indefinite). Even when multiple *XY* data sets are available, *a* and *b* are non-identifiable. Therefore, *a* or *b* must be fixed separately.

**Figure 6.**
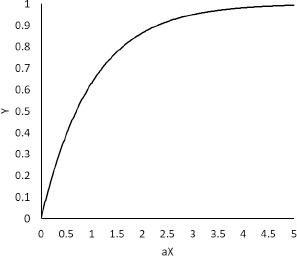
*Y* = 1 – exp (-*aX*).

The statistical identifiability can be understood by considering *Y* = 1/ (*X* + *a*). If the error of *X* is comparable to *a*, *a* cannot be reliably determined (even assuming zero error in *Y*). For example, when *X* = 100 ± 10, we cannot determine *a* smaller than 10. Preferably, *a* should be determined at *X* << *a*. Another familiar example is *Y* = 1 - exp(-*aX*) (Figure 6). In this case, *aX* should be < 1.5 if *Y* has a 20 % error. The other simple examples of parameter identifiability are shown in Supplemental Information.

### 3.3. Local middle-out approach to predict the food effect: an example

To overview the process of a local middle-out approach and understand the importance of parameter identifiability, in this section, we look at a simple example of OA PBPK modelling using a local middle-out approach and then discuss the checkpoints.

#### Step 1: Problem statement

The drug candidate is a poorly soluble non-ionizable compound. The formulation is a simple immediate-release formulation. Drug parameters are available from preclinical *in vitro* studies (Table 1). The physiological data are provided as default values.

Now, the clinical PK data in the fasted state became available after the first-in-human study (both p.o. and i.v.). The bioavailability (*F*) was 0.30 ± 0.15 (mean ± s.d.). We are asked by the managers to predict AUC in the fed state in healthy volunteers. To utilize the first-in-human clinical data, we decided to conduct a local middle-out approach.

#### Step 2: Model selection

The particle size is small enough so that the drug dissolution process does not become the rate-limiting step [70–72]. In addition, because the drug is non-ionizable, gastric dissolution would have little effect on *F*. In the case of solubility limited oral absorption, *F* can be calculated as [146]:


(1)

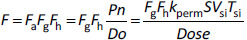



where *P*n is the permeation number (=*k*_perm_ ×*T*_si_), and *Do* is the dose number *Do* = *Dose*/ (*S* ×*V*_si_). *F*_g_ is the fraction escaping the intestinal metabolism. In this example, Eq. 1 is used as a model equation for convenience.

#### Step 3: Parameter optimization

From Eq. 1 and given data, *k*_perm_ is optimized to be 0.3 h^-1^ in the fasted state.

#### Step 4: Model validation

Using the optimized *k*_perm_ value, *F* is calculated as,



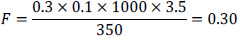



This predicted *F* value perfectly matches the clinical *F* data (= 0.30). This *k*_perm_ value is additionally validated using the independent clinical *F* data in the fasted state.

#### Step 5: Prediction

The optimized *k*_perm_ value is then used to predict *F* in the fed state. Using the solubility in FeSSIF (0.2 mg/mL), *F*_a_ is calculated as



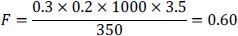



Therefore, AUC is predicted to increase twofold in the fed state.

#### Checkpoint 1: Hidden “bottom-up” prediction

Usually, we start a prediction study with a “bottom-up” approach at a preclinical stage (section 1.3). The input parameters are initially projected from the *in vitro* data. In the above case,



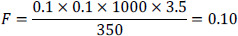



The “bottom-up” prediction resulted in a 3-fold underestimation. *k*_perm_ was increased threefold (from 0.1 to 0.3) after parameter optimization. This is not a subtle adjustment. This information alerts that there is a marked discrepancy between the *k*_perm_ values estimated from the Caco-2 data and back-calculated from the clinical *F* data (section 3.4).

#### Checkpoint 2: Subject of parameter optimization

The reason for selecting *k*_perm_ as the subject of parameter optimization is not clear (sections 1.2 to 1.4 and 3.5 to 3.7).

#### Checkpoint 3: Parameter identifiability

Eq. 1 suggests that the *k*_perm_ value is not identifiable from the provided data (*F*, *F*_h_, *Dose*, *S*, *V*_si_, and *T*_si_). *F*_g_ must be separately fixed (section 3.8 (v)). In the above calculation, *F*_g_ was unknowingly assumed to be 1 (section 1.2 to 1.4).

#### Checkpoint 4: Hidden errors

The intestinal fluid volume of 1000 mL is significantly greater than the current best estimate (most probably < 150 mL) (in the discussion below, we assume that 100 mL is realistic for the convenience of discussion) [26–29]. The optimized *k*_perm_ value carries the error in the intestinal fluid volume (e.g., 1000 mL vs 100 mL). With a more realistic intestinal fluid volume of 100 mL, the *k*_perm_ value is back-calculated to be 3.0 h^-1^, ten-fold higher than 0.3 h^-1^. This could cause a misunderstanding of the rate-limiting step for membrane permeation (section 1.4).

#### Checkpoint 5: Variation of clinical data

Considering that CV% of *F* is 50 %, it is not possible to back-calculate the *k*_perm_ value within a good confidence interval.

#### Checkpoint 6: Constancy of optimized parameter

It was implicitly assumed that the *k*_perm_ values in the fasted and fed states are the same. However, it is well known that bile micelle binding reduces the free fraction, the effective diffusion coefficient, and consequently *P*_eff_ and *k*_perm_ (section 2.7) [51,91,92,94,147,148].

#### Checkpoint 7: Model validation

The predictive power of a model cannot be validated by the same data used for parameter back-calculation (section 3.11).

#### Checkpoint 8: Prediction

After parameter optimization, the predictive range of the optimized model is limited to the parameter space of the clinical data that is used for model development (limited to interpolation with some exceptions).

In the above example, a simple model (Eq. 1) was used for convenience to illustrate the middle-out approach. In complicated PBPK modelling, these checkpoints are not so easy to recognize. However, the mathematical principle remains the same.

### 3.4. When is a local middle-out approach required?

A local middle-out approach is required when a bottom-up prediction is not satisfactory for the contest of use (section 1.3, Figure 1). The widespread use of a local middle-out approach is consistent with the results of systematic evaluation suggesting that current bottom-up OA PBPK models need significant improvement (introduction section). Discrepancies between bottom-up predictions and clinical observations may suggest opportunities to uncover unidentified mechanisms or to improve the equations and parameters of the model (Sections 1.3, 3.3 Checkpoint 4). Before parameter back-calculation, the reason for the discrepancy should be thoroughly explored (sections 1.4 and 2.7).

### 3.5. How to diagnose parameter identifiability in OA PBPK modelling

In the literature, *P*_eff_ has often been the subject of back-calculation. Therefore, the identifiability of *P*_eff_ is discussed as an example below.

S*imple analytical solutions are useful for diagnosing the parameter identifiability* (section 2.2). The *F*_a_ equation represents the relationship between the solubility, dissolution rate, and permeation of a drug to *F*_a_ [5,35,149]:


(2)

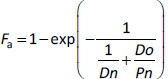



where *D*n is the product of the dissolution rate (*k*_diss_) and the intestinal transit time (*D*n = *k*_diss_ × *T*_si_). When *D*o < 1, set *D*o = 1.

When the oral absorption of a drug is limited by the dissolution rate (*D*n < *P*n/*D*o), the permeation process is statistically non-identifiable from *F*_a_ (cf. *Y* = 1/ (*X* + *a*)). In other words, for *P*_eff_ to be identifiable, the oral absorption must be permeability or solubility-permeability limited. In this case, Eq. 2 becomes:


(3)

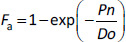



At *P*n/*D*o > 0.7, the *P*_eff_ value (that is in *P*n) is statistically non-identifiable from clinical *F*_a_ data considering the variation of clinical *F*_a_ data (cf. *Y* = 1 - exp(-*aX*), Figure 6). At *P*n/*D*o < 0.7, Eq. 3 can be approximated to [146]:


(4)

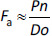



Using Eq. 4 for *F*_a_, the AUC value after oral administration can be expressed as:


(5)

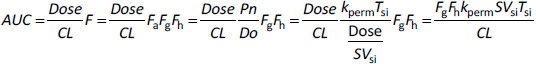



Finally,


(6)

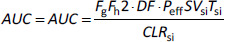



In the absence of a unique correspondence between a parameter and a *C*_p_ - time profile, it is impossible to directly quantify the physiological process that involves the parameter. When the parameters are in a multiplication relationship, they are structurally non-identifiable from clinically observed data (cf. *Y* = *abX*). As clearly represented in Eq. 6, the individual parameters related to oral drug absorption cannot be calculated solely using AUC after oral administration [19–22,33]. Therefore, an OA PBPK model is essentially overparameterized. To back-calculate *P*_eff_ from *F*_a_, the other eight parameters (*F*_g_, *F*_h_, *S*, *V*_si_, *T*_si_, *R*_si_, *DF*, and *CL*) must be fixed separately.

Parameter sensitivity analysis can be used as a support to diagnose the identifiability of parameters. However, AUC is sensitive to all the parameters on the right-hand side of Eq. 6. *Being a sensitive parameter is a necessary but not sufficient condition to be identifiable* [19–22].

A similar analysis of parameter identifiability can be performed for *C*_max_ and *T*_max_:



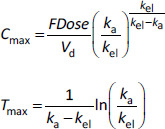



where *k*_a_ is the absorption rate constant, *k*_el_ is the elimination rate constant, and *V*_d_ is the volume of distribution. *k*_a_ can be approximated as 1/*k*_a_ = 1/*k*_diss_ + *D*o/*k*_perm_ (section 3.14).

### 3.6. What kind of parameters can be candidates for back-calculation?

Only an intermediate parameter (e.g., *P*_eff_) or an empirical scaling factor should be the candidates for the subject of parameter back-calculation from clinical PK data in a local middle-out approach (Figure 2).

Drug-intrinsic parameters, especially physicochemical properties, should not be the subject of parameter back-calculation from clinical PK data. They should be determined by *in vitro* measurements.

Physiological parameters such as the small intestinal fluid volume (*V*_si_) should not be the subject of drug-by-drug back-calculation as well, because they do not change drug-by-drug (except for drugs with gastrointestinal effects) (A global middle-out approach has been used to estimate some physiological parameters when direct measurements are not possible (section 3-15)).

Post-absorptive (systemic) PK parameters such as *CL* and *V*_d_ should be determined from i.v. *C*_p_ - time data [33] (unless *F* = 1 can be surely estimated from *in vitro* data, such as the case of BCS class I drug with low hepatic clearance). The same *CL* and *V*_d_ values should be used regardless of formulations or administration routes. Oral formulation usually does not affect systemic *CL and V*_d_ (however, it affects CL/F and *V*_d_ /F).

### 3.7. Which candidate parameter should be selected for back-calculation?

When multiple candidate parameters can equally explain the discrepancy between prediction and observation, we cannot determine which one to be selected for back-calculation solely based on the clinical data. This situation is like a checksum process. We can validate a series of numbers by checking the sum of numbers, but we cannot tell which number has the error. The selection of a parameter depends on the clinical study design and the reliability of the estimation from *in vitro* data (section 3-8, (iv)). Identifiability issues can be even more severe when there are regional differences in the gastrointestinal parameters in Eq 6.

### 3.8. How to fix the other parameters

There are several methods to fix the other parameter values.

#### (i) Clinical study using different administration routes and formulations

To reliably estimate a parameter from clinical PK data, the interference of confounding factors must be reduced as much as possible. A specific clinical study design has been employed to eliminate the confounding factors for each parameter (Table 2) (see also (3.13)). For example, systemic clearance (*CL*) can be obtained from i.v. data. An i.v. administration eliminates the oral absorption process (*F* = 1), so that *CL* become identifiable from AUC (cf. *CL* = *Dose*/AUC). *V*_d_ and *F*_h_ can also be calculated from the i.v. data [150]. An oral solution formulation can be used to eliminate *Dn* from Eq. 2 (1/Dn becomes negligible), and *S* and *V*_si_ from Eq. 6 (mathematically, by fixing *Do* = 1).

It is not known whether parameter back-calculation from the oral *C*_p_ - time data after the administration of solid dosage forms can be accurate. Theoretically, the dissolution processes can be decomposed from the permeation process by using the *C*_p_ - time data from solution formulations [154]. However, this deconvolution process often becomes unstable due to the variation of the data. In addition, there has been no systematic evaluation of *P*_eff_ back-calculation from the oral *C*_p_ - time data after the administration of solid dosage forms. Unfortunately, in the literature, *P*_eff_ has been back-calculated even in the absence of i.v. data in many cases. The credibility of such *P*_eff_ back-calculation is at least questionable (section 3.9).

#### (ii) Experimentally measured physiological parameters

Experimentally measured physiological parameters can be obtained from the literature (*V*_si_, *T*_si_, and *R*_si_ in Eq. 6). However, it should be noted that some of the physiological parameters reported in the literature have large variations between subjects and between occasions.

#### (iii) Physiological parameters estimated by a global middle-out approach

The global middle-out approach has been used to back-estimate an unknown physiological parameter from clinical PK data (*DF* in Eq. 6, section 3.15).

#### (iv) Preclinical *in silico*, *in vitro*, and *in vivo* data

Preclinical *in silico*, *in vitro*, and *in vivo* data can be used to fix a parameter if their predictability is sufficient considering the contest of use (COU) (section 1.2). The *in vitro* equilibrium solubility in biorelevant media such as FaSSIF and FeSSIF has often been assumed to be *in vivo* predictive. However, it should be noted that these are simplified artificial fluids, not actual intestinal fluids. FaSSIF and FeSSIF are very good model fluids, yet, the solubility values do not always accurately reflect the real *in vivo* values [155]. In addition, the solubility data could be inaccurate when inappropriately measured [109].

Current *in vitro*-*in vivo* extrapolation (IVIVE) and allometric scaling for *CL* and *V*_d_ is not sufficiently accurate for the COUs of OA PBPK modelling in the late drug development stage [7].

*In silico* models for the physicochemical properties and other ADME properties of a drug are not so accurate as to be used for PBPK modelling [120] (except for molecular diffusion coefficients [156, 157]).

#### (v) Hypothesis generation

The *P*_eff_ value is still not identifiable after fixing *S*, *V*_si_, *T*_si_, *R*_si_, *DF*, *CL*, and *F*_h_. Hypothesis generation is required to estimate *F*_g_ [14]. Hypothesis generation may include IVIVE, but in a less qualitative manner. Hypothesis generation about the negligibility of a parameter would be most credible and useful because it can reduce the interference from the parameter for back-calculation. The credibility of a hypothesis can be improved by combining various *in vitro*, preclinical *in vivo*, and clinical observations. For example, when *in vitro* data suggest that a drug is not a substrate of intestinal metabolic enzymes, *F*_g_ = 1 (no gut wall metabolism) can be a plausible hypothesis. Low intrinsic hepatic clearance (< 100 mL/min/kg)[158], the lack of metabolites, and the lack of clinical grapefruit–drug interaction can further support the hypothesis of *F*_g_ = 1.

### 3.9. A good example of credible parameter back-calculation

Sjögren et al. reported that *P*_eff_ can be credibly identifiable by the deconvolution of the *C*_p_ - time profiles after intraintestinal bolus administration as a solution, using i.v. disposition data [151]. They pointed out that the *C*_p_ - time profiles after an oral administration may not be suitable for *P*_eff_ estimation due to possible interference of confounding factors. They used a solution formulation to eliminate the uncertainty in *S*, *V*_si_, and the effect of dissolution processes. Intra-intestinal administration was used to eliminate the effect of gastric emptying. The i.v data was used to calculate *CL* and *F*_h_. Hypothesis generation was used for *F*_g_. The basic concept of their approach is shown in Figure 8 (They used the deconvolution method, but it was simplified to AUC calculation to explain the concept).

### 3.10. Accumulation of errors

After parameter back-calculation, the simulation curve would show perfect fitting to the observed *C*_p_ -time data that had been used for parameter back-calculation. However, this perfect fitting does not imply the validity of all parameters and model equations.

*A back-calculated parameter inherits the errors of the other parameters* (see Checkpoint 4)*.* In the case of Eq. 6, the errors in nine separately fixed parameters (*V*_si_, *T*_si_, *R*_si_, *DF*, *S*, *CL*, *F*_g_, *F*_h_, and clinical AUC) exponentially accumulate in *P*_eff_ (sections 2.2, 2.8). Even if each parameter has only a small error of 20 % (1.2-fold), the total error can become 5.2-fold (= 1.2^9^). To back-calculate *P*_eff_ within less than 20 % error, each parameter must have less than 2 % error. Any experimentalist knows that this is not possible. Furthermore, back-calculating one parameter hides the errors of the other parameters and model equations (section 3.4). *P*_eff_ itself can show high inter-subject variability [159].

### 3.11. How to validate the predictive power of a PBPK model after parameter back-calculation

In a local middle-out approach, the predictive power needs to be validated for each drug after parameter back-calculation. The parameter back-calculated in one clinical condition is not necessarily valid in the other clinical condition (section 3.3, checkpoint 6). The predictive power of a model cannot be validated by the same data used for parameter back-calculation, because it is self-referencing. Therefore, the predictive power needs to be validated using other clinical PK studies of the context of use (COU) (“cross-validation”). The PK data under clinical conditions where some of the same pathways in the system are perturbed as in the COU can be employed for validation [10,21]. In the case of food effect prediction, the optimized model should be validated by the *C*_p_ - time data under a clinical condition in which the pathways of oral absorption are perturbed by the same factor for the food effect. In other words, the food effect prediction must be validated by a clinical food effect study.

The clinical PK data in the same clinical condition cannot be used for validation, even if it is independently determined, because the *C*_p_ - time profiles are expected to be similar under the same clinical condition (this is a kind of “leakage” in the cross-validation process). Multiple-dose PK data under the same clinical condition cannot be used for validation (unless the context of use is the prediction of metabolic enzyme induction or mechanism-based inhibition in the intestine after multiple-dose PK).

### 3.12. “Confirm and refine“ strategy in drug discovery and development

The quality and quantity of experimental data available for PBPK modelling increase as a research project proceeds in drug discovery and development. Therefore, it would be beneficial to utilize these data for PBPK modelling (section 2.8).

Each module in an OA PBPK model can be confirmed by comparison with corresponding *in vitro* experimental data covering a wider range than *in vivo* conditions. For example, the solubility model should be validated by an experimental pH-solubility profile in the range of pH 1.0 to 8.0 to cover *in vivo* gastrointestinal conditions. The dissolution model (the Nernst-Noyes-Whitney equation) can be confirmed by comparison with dissolution test data. If the prediction result deviated from the experimental observation, an empirical correction parameter can be introduced, like the z factor for each dissolution condition [81].

Similarly, *in vivo* animal PK data can be used to inform the confidence level of a PBPK model. However, in this case, the refinement (back-calculation) process requires the same cautions as the local middle-out approach. In addition, there may be species differences in the back-calculated parameter.

First-in-human PK data can be used to inform the confidence level of a PBPK model in humans. The PK data at a low dose (dose number << 1) can be used to evaluate the oral absorption process without interference from the dissolution rate and solubility. In the case of high permeability drugs, the low dose PK data may provide information regarding systemic clearance and volume of distribution to some extent (but i.v. PK data is preferable). The confidence level of solubility and permeability values (as Pn/Do, Eq. 2) may also be evaluated by the dose sub-proportionality of AUC (so that relative bioavailability between low and high doses). However, as discussed above, it is not easy to accurately back-calculate (refine) a parameter from *in vivo* oral PK data. Extreme caution should be exercised when using a middle-out approach.

### 3.13. What is the difference between metabolic DDI and food effect predictions?

A local middle-out approach has been successfully used to predict metabolic DDI by PBPK modelling [12,160,161]. Because it is practically impossible to clinically evaluate all DDI combinations, DDI prediction by a local middle-out approach would be of great value. In the local middle-out approach for DDI, to identify the fraction of metabolic clearance (*f_m_*) (e.g., a CYP isozyme), a specific inhibitor has been used in a clinical PK study (section 3.8 (i)). In the following, the concept of DDI prediction by the local middle-out approach is briefly explained (see [12,160,161] for details)*.*

In the case of i.v. dosing (or *F* = 1 for oral dosing), *CL* and AUC in the absence of an inhibitor can be expressed as:



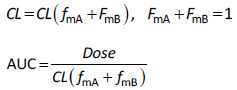



where *f*_mA_ and *f*_mB_ are the fractions of metabolic clearance pathways (A and B, respectively). The *f*_mB_ can be calculated from the AUC ratio in the absence (AUC_no-inhibition_) and presence (AUC_inhibition_, *f*_mB_ = 0) of a specific strong inhibitor as:



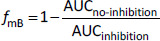



The inhibitor should specifically inhibit only pathway B and there is no other underlying clearance mechanism (e.g. renal clearance) or uptake/efflux transporter in interplay which can be influenced by the inhibitor. In this case, the *f*_mB_ value is identifiable from the AUC ratio. This equation also suggests that the AUC ratio is predictable without using any complex PBPK modelling. A simple prediction scheme for metabolic DDI (AUC ratio) has been proposed and thoroughly validated using a large number of clinical DDI data (section 2.8) [67,162–164].

However, a similar approach cannot be simply applied to the food effect prediction, because a food intake simultaneously affects various processes of oral drug absorption (section 2.7) [165].

### 3.14. What is the difference between PBPK and compartmental PK models?

In pharmacokinetics, the compartmental PK model is widely used. Although both compartmental PK models and PBPK models belong to mathematical models, their applications are different.

**Figure 7.**
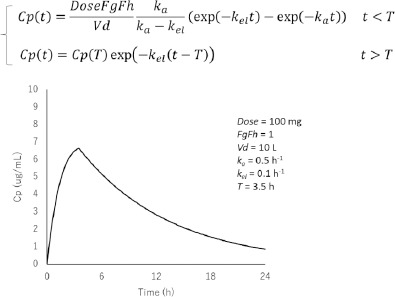
An oral one-compartment model with an finite absorption time of *T* = 3.5 h [86,166]. In many cases, an oral *C*_p_ - time profile can be summarized into three parameters, *k*_a_, the elimination rate constant (*k*_el_), and the lumped values of the volume of distribution (*V*_d_), *F*_g_, and *F*_h_ (*V*_d_ /*F*_g_*F*_h_). *T*_max_, *C*_max_, and AUC are all described by using only one absorption parameter (*k*_a_) that represents the oral absorption of a drug. Therefore, the oral absorption of a drug can be well described by fitting one of many conjugated parameters in a complex OA PBPK model (the degree of freedom is zero). From the *C*_p_ -time profile after oral administration, only the composite parameter of *V*_d_/*F*_g_*F*_h_ is identifiable. *An i.v. data is required to fix V*_d_ *(and CL)* [33].

**Figure 8.**
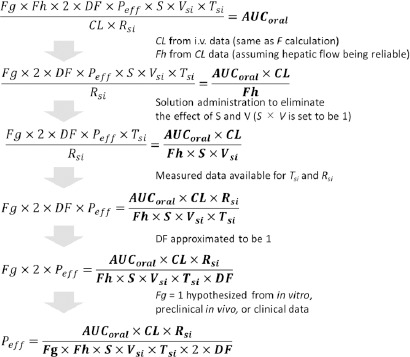
*P*_eff_ calculation scheme using a set of clinical data, literature data, and hypothesis generation. Each parameter is fixed step-by-step. The parameters after the evaluation are moved to the right side of the equation and shown in bold type. Eq.6 is used to understand the concept of back-calculation. Sjögren et al. used the deconvolution method [151], but the basic concept of parameter identification is the same (the deconvolution method can eliminate the uncertainty in *T*_si_).

In compartmental PK models, all parameters are calculated from clinical PK data (“top-down” approach) (section 3.1). Because the compartmental PK model is empirical, it is used following the good practice of empirical modelling (section 3.1). To avoid overfitting (section 2.2, Figure 3), the Akaike information criterion has been used to select the number of compartments appropriately [69]. For an i.v. PK model, the number of compartments is set to be one or two in most cases (2 and 4 parameters, respectively). For an oral PK model, only three parameters, that is, *k*_el_, *k*_a_, and *V*_d_ /F are used in most cases (Figure 7).

In contrast, a PBPK model consists of dozens to hundreds of parameters (section 2.1) [37]. All these parameters cannot be identified from the *C*_p_ -time profile alone (section 3.8).

### 3.15. Global middle-out approach (system parameter estimation)

In PBPK modelling, it is preferable to use experimentally measured physiological parameters. However, some of the physiological parameters are not available. In such a case, a global middle-out approach has been used to back estimate a physiological parameter from the multiple PK data of multiple drugs (cf. A local middle-out approach is on a drug-by-drug basis) (section 3.1) [83,90,129,145,167]. In this approach, the following three points are usually carefully considered to ensure parameter identifiability and avoid overfitting: (i) enough number of data covering a wide range of dependent and independent variables, (ii) *in vivo* PK data that is sensitive to the physiological parameter (and not sensitive to the other parameters), (iii) comparison with directly or indirectly measured values.

For example, the volume of the small intestinal fluid (*V*_si_) available for drug dissolution has not been clear, because only free water can be directly measured by MRI [28,29]. The *V*_si_ value available for drug dissolution was back-calculated from the clinical PK data of several low solubility drugs at various dose strengths covering Do < 1 and Do > 1 [83,167]. The estimated *V*_si_ values (130 mL [83] and 116 mL [167]) were about 1.5 to 2-fold larger than the average free water volume directly measured by various techniques [26,28,29]. *DF* in Eq. 6 was obtained from the relationship between the clinical *F*_a_ and *P*_eff_ values of about 20 high solubility drugs [55] (cf. *F*_a_ = 1 – exp (- 2*DF*/*R*_si_ x *P*_eff_ x *T*_si_).

## Conclusion: strategy to improve oral absorption physiologically-based pharmacokinetic modelling

### Multidisciplinary collaboration

A good collaboration of pharmacokinetic, physical chemistry, formulation, and physiology experts is required to improve oral absorption (OA) physiologically-based pharmacokinetic (PBPK) modelling. There are many opportunities for both experimentalists and modellers to take advantage of collaborative works in this field [32].

### The critical role of physical chemistry in OA-PBPK modelling

The importance of physical chemistry in PBPK modelling has often been overlooked (section 2.7). Physical chemistry plays a central role in oral drug absorption [86]. In addition, physical chemistry is also important for hepatic clearance, renal clearance, and tissue distribution (including the brain) [85,168–176]. A recent survey suggested that a poor understanding of physical chemistry is one of the reasons for the prediction failure of OA PBPK modelling [6]. A good understanding of the chemical equilibrium [177], nucleation theory [115,178], and fluid dynamics (including mass transport) [172,173,179] is required in OA PBPK modelling. Fortunately, physical chemists are generally well trained in mathematics and mechanistic modelling. They are familiar with the concept of parameter identifiability.

### The critical role of physiological parameters in OA PBPK modelling

Similarly, an in-depth understanding of gastrointestinal physiology is critically important [165,180,181]. Physiological parameters reported in the literature have large variations, for example, in gastric pH, intestinal pH, fluid volumes, bile concentration, and buffer capacity [27,182]. It is a combination of experimental uncertainty, true inter-subject variability and true inter-occasion variability. The buffer capacity of compendial dissolution media (phosphate buffer) is markedly higher than the real intestinal fluid (bicarbonate buffer), affecting the dissolution profiles of drugs [61,183–186]. The intestinal fluid volume (*V*_si_) would be much smaller than originally thought [25–29] (note that a *V*_si_ value (212 mL) had already been reported as early as 1957) [26,27]. The gastric and intestinal pHs of dogs are significantly different from those in humans [50,187–190].

### Harmonization

In the future, it is desirable to harmonize physiological parameters and model equations for regulatory submission (section 1.2 and 2.1). In addition, drug intrinsic parameters such as *S*_0_, *P*_trans0_, and *K*_bm_ (section 2.1), should be obtained using harmonized procedures [109]. Currently, the prediction characteristics of commercial software products vary from product to product [5]. If two software products predict different outcomes, which one should be used for regulatory purposes? In the field of biopharmaceutics, the procedures and conditions for dissolution tests [191,192], in vitro – in vivo correlation [193,194], the biowaiver scheme [195], etc. have been harmonized and standardized. Similarly, OA PBPK modelling should be harmonized based on proper model evaluation in the future (section 2.4). We may also need a good simulation practice when PBPK modelling is used to waive a clinical study [35,36].

### Not to be lost in modelling and simulation

***“The greatest enemy of knowledge is not ignorance; it is the illusion of knowledge”***

Daniel Boorstin/ Stephen Hawking

When the “bottom-up” simulation deviates from the clinical data, simply press the "optimize" button and the monitor will display a simulation curve that exactly matches the clinical plasma concentration (*C*_p_) -time data. If we continue publishing this as a successful "prediction", it will eventually cause the illusion of a prediction paradise.

We must exert extreme caution not to be lost in modelling and simulation. Model equations, physiological parameters, and drug parameters must be disclosed to ensure proper peer-review and reproducibility. A systematic evaluation of predictive power is required to avoid publication bias. When a middle-out approach is pursued, the fitted *C*_p_ - time curve must be labelled as "fitted" (NOT "predicted"). Parameter identifiability should be carefully considered. The optimized model must be validated using independent clinical PK data of the context of use. Finally, and most importantly, pharmacokinetic, physical chemistry, formulation, and physiology experts should work together so that not to get lost in modelling and simulation.

As mentioned in the introduction, when used correctly, OA PBPK modeling will be an excellent tool for understanding and predicting the oral absorption of a drug. This article will hopefully enhance the science of OA PBPK modelling in the future*.*



## Figures and Tables

**Table 1.** Drug and physiological parameters (example)

Parameter	Value	Comments
*Drug parameter*		
Permeation rate constant (*k*_perm_)	0.1 h^-1^	From Caco-2 data (*P*_eff_ = *a P*_app_^*b*^)
Effective solubility (*S*)	0.1 mg/mL 0.2 mg/mL	Fasted state simulated intestinal fluid (FaSSIF) Fed state simulated intestinal fluid (FeSSIF)
Dose strength (*Dose*)	350 mg	
Particle size	5 μm	
Diffusion coefficient	6 × 10^-6^ cm^2^/s	
*Physiological parameter*		
Small intestinal fluid volume (*V*_si_)	1000 mL	Default (see Checkpoint 4)
Small intestinal transit time (*T*_si_)	3.5 h	Default
Small intestinal radius (*R*_si_)	1.5 cm	Default
Degree of flatness (*DF*)	1.7	Default
Fraction escaping first pass hepatic metabolism (*F*_h_)	1	Calculated from i.v. *CL*_h_ and hepatic flow

**Table 2.** The parameters and the clinical study design

Parameter	Clinical study design	Reference
*CL*, *V*_d_	i.v. administration	-^a^
*P*_eff_	Site-specific solution administration and i.v. administration	[151]
Precipitation rate	Site-specific solution administration and sampling	[152][153]

^a^ See any pharmacokinetic textbook
