# Supplementary material for: Lost in modelling and simulation?
Source: ADMET DMPK. 2021 Mar 22;9(2):75–109. doi: 10.5599/admet.923 (PMC8920108; doi:10.5599/admet.923)
Supplement: Supplementary file 1 [file admet-9-923-S1.pdf]

Supporting Information for

Lost in modelling and simulation?

ADMET and DMPK (2020) doi: **https://doi.org/10.5599/admet.923**

Mathematical concept of structural identifiability (indefinability)

Structural identifiability is critical to ensure that the unknown model parameters are uniquely identifiable from a specified experimental observation. The mathematical concept of structural identifiability is explained in Figure S1. Because PBPK models are a mathematical model, the problem of structural identifiability cannot be avoided based on mathematical principle.

$Y = abX$	The parameter *a* and *b* are nonidentifiable (indefinite) from any *X* and *Y* data. Only identifiable as a composite parameter (= *ab*).
$Y = aX + b$	The parameter *a* and *b* can be identified from greater than or equal to two pairs of *X* and *Y* data.
$Y = aX^2 + bX + c$	The parameter *a, b,* and *c* can be identified from greater than or equal to three pairs of *X* and *Y* data.<br>In general, the number of X and Y pairs must be greater than or equal to the number of parameters. To avoid over-learning, it is preferable to use a smaller number of parameters with a larger number of XY pairs.
$Y = X + a$<br>$Y = \frac{1}{X + a}$	Mathematically, the parameter *a* is identifiable from one pair of *X* and *Y* data. However, when the *X* data have an experimental error comparable to the absolute value of *a*, the parameter *a* cannot be practically identified.

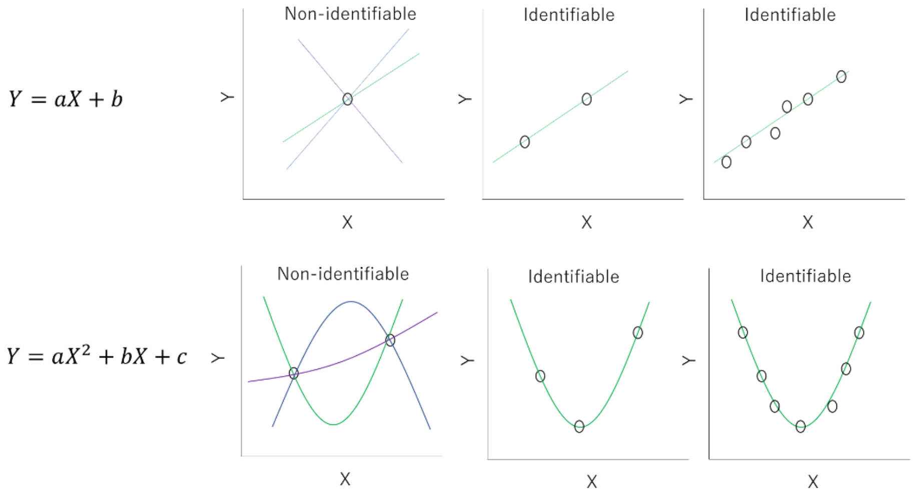

Figure S1. Structural and numerical identifiability
